# Degradation of macroalgal detritus in shallow coastal Antarctic sediments

**DOI:** 10.1002/lno.11125

**Published:** 2019-02-05

**Authors:** U. Braeckman, F. Pasotti, S. Vázquez, K. Zacher, R. Hoffmann, M. Elvert, H. Marchant, C. Buckner, M. L. Quartino, W. Mác Cormack, K. Soetaert, F. Wenzhöfer, A. Vanreusel

**Affiliations:** ^1^ Marine Biology Research Group Ghent University Ghent Belgium; ^2^ HGF‐MPG Group for Deep Sea Ecology and Technology, Alfred Wegener Institute Helmholtz Center for Polar and Marine Research, Bremerhaven and Max Planck Institute for Marine Microbiology Bremen Germany; ^3^ Cátedra de Biotecnología, Facultad de Farmacia y Bioquímica Universidad de Buenos Aires, NANOBIOTEC UBA‐CONICET Buenos Aires Argentina; ^4^ Functional Ecology, Alfred Wegener Institute Helmholtz Center for Polar and Marine Research Bremerhaven Germany; ^5^ MARUM Center for Marine Environmental Sciences and Department of Geosciences University of Bremen Bremen Germany; ^6^ Biogeochemistry Group Max Planck Institute for Marine Microbiology Bremen Germany; ^7^ Instituto Antártico Argentino, Coastal Biology Department Buenos Aires Argentina; ^8^ Museo Argentino de Ciencias Naturales Bernardino Rivadavia Buenos Aires Argentina; ^9^ NIOZ Yerseke, Estuarine and Delta Studies and Utrecht University The Netherlands

## Abstract

Glaciers along the western Antarctic Peninsula are retreating at unprecedented rates, opening up sublittoral rocky substrate for colonization by marine organisms such as macroalgae. When macroalgae are physically detached due to storms or erosion, their fragments can accumulate in seabed hollows, where they can be grazed upon by herbivores or be degraded microbially or be sequestered. To understand the fate of the increasing amount of macroalgal detritus in Antarctic shallow subtidal sediments, a mesocosm experiment was conducted to track ^13^C‐ and ^15^N‐labeled macroalgal detritus into the benthic bacterial, meiofaunal, and macrofaunal biomass and respiration of sediments from Potter Cove (King George Island). We compared the degradation pathways of two macroalgae species: one considered palatable for herbivores (the red algae Palmaria decipiens) and other considered nonpalatable for herbivores (the brown algae Desmarestia anceps). The carbon from Palmaria  was recycled at a higher rate than that of *Desmarestia*, with herbivores such as amphipods playing a stronger role in the early degradation process of the *Palmaria* fragments and the microbial community taking over at a later stage. In contrast, *Desmarestia* was more buried in the subsurface sediments, stimulating subsurface bacterial degradation. Macrofauna probably relied indirectly on *Desmarestia* carbon, recycled by bacteria and microphytobenthos. The efficient cycling of the nutrients and carbon from the macroalgae supports a positive feedback loop among bacteria, microphytobenthos, and meiofaunal and macrofaunal grazers, resulting in longer term retention of macroalgal nutrients in the sediment, hence creating a food bank for the benthos.

The western Antarctic Peninsula is one of the fastest warming areas on Earth (Ducklow et al. 2007). As a result, its glaciers are melting and retreating at unprecedented rates (Rückamp et al. 2011; Cook et al. 2016). The retreat of glaciers opens up new habitat for marine benthic organisms (e.g., Lagger et al. 2018), such as sublittoral rocky substrates that are increasingly colonized by macroalgae (Quartino et al. 2013; Mystikou et al. 2014; Campana et al. 2018). Macroalgal communities play an important role in the Antarctic coastal ecosystem. They dominate shallow benthic communities on hard substrates along the western Antarctic Peninsula, often covering > 80% of the bottom, with standing biomass levels comparable to temperate kelp forests (Wiencke and Amsler 2012). A global average of 82% of the local primary production from kelp is estimated to enter the detrital food web where it can be exported to adjacent communities (Krumhansl and Scheibling 2012).

The fate of detached macroalgal fragments is not well‐known. Due to storms, erosion, or ice movement, macroalgal fragments can be detached, sink and accumulate in seabed hollows, and be washed ashore or drift in the sea (Klöser et al. 1996). If not grazed directly by herbivores, macroalgal fragments decompose, releasing particulate and dissolved organic matter (DOM) into the environment (Hanisak 1993; Barrón et al. 2014). The seaweed decomposition process thereby depends on the place where it occurs (Zielinski 1981). In the water column, physical agents like waves and currents seem to play a more important role in the macroalgae breakdown than microbial degradation, which results in slow degradation rates (Brouwer 1996), although an increase in numbers of heterotrophic bacteria has been observed during the decomposition process of brown macroalgae in the water column (Quartino et al. 2015). Only a small (< 3%) fraction of the macroalgal production strands ashore depending on wind direction and coastal orientation, such as observed on Deception Island (Lastra et al. 2014). On shores, the mix of dead macroalgal remains, coastal sediments, and seal excrements can facilitate the development of the microorganisms, increasing remineralization rates (Zielinski 1981) and the establishment of herbivorous (Lastra et al. 2014) and bacterivorous communities (Alkemade and Van Rijswijk 1993; Urban‐Malinga and Burska 2009). On bare subtidal sediment, algal debris can have three fates: (1) assimilation into new biomass, either microbial or of larger fauna. For example, in deep Arctic fjord sediments, macroalgal detritus is an important food source for suspension‐feeding bivalves (Renaud et al. 2015), shrimp and amphipods (Ramirez‐Llodra et al. 2016). (2) The assimilated macroalgal carbon can be respired and the CO_2_ returned to the atmosphere. (3) Macroalgae also contain refractory material (typically 0–10%; Nielsen et al. 2004) that can become buried below the sediment surface, locking it away from exchange with the atmosphere and contributing as such to blue carbon storage (Hill et al. 2015; Trevathan‐Tackett et al. 2015; Krause‐Jensen and Duarte 2016). The degradation rates and pathways of macroalgal detritus in subtidal sediments of the Antarctic have, however, not been quantified so far.

Potter Cove, a shallow coastal subantarctic bay on King George Island/Isla 25 de Mayo, South Shetland Islands, harbors a complex macroalgal community with high biomass production. Until the end of the last century, the macroalgae distribution was restricted to the outer, hard bottom coastal areas (Klöser et al. 1996; Quartino et al. 2005). In the last two decades, the retreat of the Fourcade glacier (Rückamp et al. 2011) triggered the colonization of the inner cove by macroalgae (Quartino et al. 2013; Campana et al. 2018). As phytoplankton biomass is generally low in Potter Cove (Schloss and Ferreyra 2002), both macroalgae and microphytobenthos have been proposed to constitute an important food supply for benthic organisms (Iken 1999; Quartino and Boraso de Zaixso 2008; Hoffmann et al., 2018). As most brown macroalgae produce secondary metabolites used as a chemical defense against herbivory (Amsler et al. 2014), direct herbivory by amphipods on especially brown macroalgae is considered unlikely (Aumack et al. 2017). However, some macrofauna, such as gastropods, annelids, crustaceans, echinoderms, and fish seem to graze on macroalgae themselves (McClintock [Link]; Iken et al. 1997, 1998, 1999; Iken 1999). Amphipods, for instance, have been observed to prefer macroalgal detritus over fresh thalli (Reichardt and Dieckmann 1985). Macroalgal detritus (small leave fragments) has been found in the diet of filter and suspension‐feeding ascidians (Tatián et al. 2004, 2008), and the deposit‐feeding bivalve *Aequiyoldia eightsii* is also thought to consume macroalgal detritus (Dunton 2001; Corbisier et al. 2004).

A large contribution of carbon entering nearshore communities is from brown macroalgal detritus (Dunton 2001; Corbisier et al. 2004). Even in cold environments with oxygen‐rich waters such as those present in Potter Cove, macroalgal decay in sediments can locally lead to hypoxic conditions in the surface sediments (Pasotti et al. 2014; Hoffmann et al. 2018). With increases in water temperature as a result of global warming, the microbial degradation of macroalgal detritus could be intensified, increasing respiration and the release of nutrients (Lastra et al. 2018). This can have a severe influence on the oxygen availability for sediment‐inhabiting fauna and the nutrient content at the sediment–water interface. Hardison et al. (2010) conducted a study on the microbial degradation of the red algae *Gracilaria* and the recycling of the macroalgal nutrients by benthic microalgae in a temperate shallow lagoon. They identified a strong link between heterotrophic bacteria decomposing the macroalgal detritus and benthic microalgae incorporating the released nutrients from the macroalgae. The sediment acted as a sink for nutrients resulting from the macroalgae decomposition, preventing further eutrophication of the water column (Hardison et al. 2010). As abundant microphytobenthos is present in the euphotic sediments of Potter Cove (Al‐Handal and Wulff 2008), we also expect a strong interaction between bacteria and microphytobenthos induced by the degradation of macroalgae in this area.

To understand the fate of the increasingly accumulating macroalgal detritus in Antarctic shallow subtidal sediments, we conducted a mesocosm experiment in which we used a dual stable isotope labeling approach to track macroalgal C and N into the sediment microbial, meiofaunal, and macrofaunal biomass and respiration after a simulated deposition of dead macroalgae fragments. We compared the degradation pathways of two macroalgae species: one species considered palatable for herbivores (the red algae *Palmaria decipiens* [Reinsch] R.W. Ricker) and other considered unpalatable for herbivores (the brown algae *Desmarestia anceps* Montagne) (Amsler et al. 2005). We hypothesized that the degradation of the more palatable *P. decipiens* would be (1) faster and (2) more herbivore driven as compared to the degradation of the unpalatable *D. anceps* that would mainly be degraded by microbes.

## *Materials and methods*

### Experimental setup

Twenty sediment cores of 10 cm internal diameter and 25 cm height were randomly collected by SCUBA divers in February 2015 at the North coast of Potter Cove (62°13′32.6″S and 58°40′03.7″W) at 18 m water depth. This location is close to the rocky shore covered with macroalgae and is situated in the main macroalgae deposition and decomposition area of Potter Cove (Quartino et al. 2008). The sediment at the study site was muddy sand with a porosity of 0.78 in the upper centimeter (63% sand; 36% mud; Pasotti et al. 2015a,*b*). Cores were brought back to the lab within 1 h after collection, about half filled with sediment (10 cm) and topped up with in situ seawater filtered on a 32 *μ*m mesh. The cores were submerged and randomly allocated in a temperature‐controlled water bath (1–2°C; in situ temperature). Before the start of the experiment, five cores were sliced to determine background pigment concentrations and organic carbon and nitrogen content (see below) and the natural stable isotopic signature of the sediment community (bacteria, meiofauna, and macrofauna).

Two types of macroalgae were added in a freeze‐dried, shredded state (a few millimeter long) to the cores (Supporting Information Fig. S1): five cores received 2.55 g C_org_ m^−2^ (0.21 g N m^−2^) *D. anceps*, five cores received 2.55 g C_org_ m^−2^ (0.36 g N m^−2^) *P. decipiens*, and five cores did not receive macroalgae (control). These are considered as the three treatments and will be further referred to as “*Desmarestia*,” “*Palmaria*,” and “control.” The molar C : N ratio of the algae was 14 for *Desmarestia* and 8 for *Palmaria*. The macroalgae had previously been grown from gametophytic stock cultures (for details, see e.g., Zacher et al. [2016]). After the development of young sporophytes, both species were cultured in NaH^13^CO_3_‐ and ^15^NH_4_Cl‐labeled medium for 2 weeks, of which the labeling success was 11 (*Desmarestia*) to 17 (*Palmaria*) at% ^13^C and 38 (*Desmarestia*) to 34 (*Palmaria*) at% ^15^N. When sufficient biomass was available, the macroalgae were harvested, washed to remove the labeled dissolved inorganic carbon (DIC) and dissolved inorganic nitrogen (DIN), and freeze dried.

The fragments settled within 24 h on the sediment surface. The cores were maintained at a 17 : 7 h day : night cycle of a constant 10 *μ*mol photons m^−2^ s^−1^ (according to an average in situ irradiance measured with PAR sensor [Odyssey Photosynthetic Irradiance Recording System, Data Flow Systems] at 18 m water depth and 0.5 m above the seafloor) at in situ temperature (2°C) and were aerated continuously. The overlying water was carefully replaced every second day to avoid accumulation of metabolites. The experiment ran in total for 26 d. Unfortunately, one core to which *Desmarestia* was added was lost. Therefore, there are no measurements for the *Desmarestia* treatment after 14 d, which is indicated with “n.d.” in the results.

### Flux measurements

At five time points during the course of the experiment (every 6–7 d), three cores (one from each treatment) were closed air‐tight and incubated to measure the sediment–water exchange of ^13^C‐DIC and ^15^N‐DIN, total DIC and DIN, and O_2_ consumption. Cores were incubated in the dark for about 12 h, ensuring O_2_ concentration never dropped below 50% of saturation. Afterward, the cores were reopened, aerated, and sacrificed for sediment sampling the next day (~ 12 h after the end of the incubations).

During the incubations, oxygen concentration was monitored continuously with a PyroScience needle‐type oxygen sensor (50 *μ*m tip size, response time < 2 s, and accuracy ± 0.02%) attached in the core lid. Discrete samples of the overlying water for ^13^C‐DIC and ^15^N‐DIN (12 mL), total DIC (2 mL), and DIN (5 mL) measurements were taken in duplicate at the start of the incubation, after 6 h and at the end of the incubation. Fluxes were calculated from the slope of significant linear regressions after correcting for dilution at time step 2 (all parameters except O_2_) or from the overall linear decrease in oxygen concentration. Due to the limitations in diving time and lab facilities, we opted for more time intervals rather than replicates, assuming that consistent trends over time support the reliability of our observations. This approach has been proven valuable in earlier studies with logistic constraints, especially in extreme environments (Guilini et al. 2010).

### Sediment subsampling

Before sediment subsampling, the overlying water was carefully siphoned off. The upper 5 cm sediment of each core was sliced in 1 cm horizons. Each horizon was homogenized and subsampled for the analysis of pigments (5 mL, stored at −80°C), lipids (10 mL, stored at −80°C), and organic carbon and nitrogen (5 mL, stored at −20°C). The rest of the sediment was frozen at −20°C for later stable isotope analysis of meiofauna and macrofauna.

### Laboratory analyses

#### *Water samples*

##### ^13^C‐DIC analyses

A 1.5 mL water subsample was transferred to a 12 mL glass vial (Exetainers; Labco) and degassed with helium. Hundred fifty microliters of 20% H_3_PO_4_ was added, and the samples were left overnight so that the DIC in solution would fill the headspace in the form of CO_2_. This headspace was then measured eight times, and the stable isotope ratio (^13^C/^12^C) was determined via a Gas Bench II (Thermo Electron) coupled to an isotope‐ratio mass spectrometry (IRMS; Thermo Quest Delta Plus, Thermo Electron). CO_2_ was used as a reference gas, and bicarbonate standards with concentrations similar to that of the samples were also added as reference. The standard deviation of the measurements was < 0.001 at%. To determine carbon respiration, the change in the stable isotope ratio (excess at% ^13^C) from the initial time point was multiplied by the total DIC concentration measured by flow injection analysis with conductivity detection (Hall and Aller 1992) with a precision better than 2%.

##### ^15^N‐DIN analyses

Subsamples (4 mL) were transferred to 6 mL glass vials (Exetainers; Labco) and degassed with helium. ^15^NH_4_^+^ was oxidized with hypobromite to N_2_ (Warembourg 1993; Preisler et al. 2007). A second set of 4.5 mL subsamples was also transferred to exetainers, and ^15^NO_x_^−^ (^15^NO_2_^−^ + ^15^NO_3_^−^) concentrations were determined after conversion to N_2_. ^15^NO_3_^−^ was reduced to ^15^NO_2_^−^ using spongy cadmium, followed by ^15^NO_2_^−^ conversion to N_2_ using sulfamic acid (Füssel et al. 2012). The stable isotope ratios of ^28^N_2_, ^29^N_2_, and ^30^N_2_ were analyzed on a gas chromatography (GC)‐IRMS (VG Optima, Micromass). The standard deviation of the measurements was < 0.001 at%. Concentrations and rates of ^29^N_2_ and ^30^N_2_ production were calculated from the excess relative to air, as explained in detail in Holtappels et al. (2011), and the efficiency of ^15^NO_x_^−^ or ^15^NH_4_^+^ conversion to N_2_ was verified (> 85%) using known concentrations of ^15^NH_4_^+^ or ^15^NO_3_^−^. We did not correct for conversion efficiency in order to be conservative.

#### *Sediment samples*

To quantify uncharacterized particulate organic carbon (POC) and nitrogen (PN) in the sediment (i.e., bulk POC and PN, possibly containing small macroalgae fragments, bacteria, microphytobenthos, and meiofauna), oven‐dried sediment samples were decalcified overnight with the fumes of 37% HCl in a desiccator. Prepared samples were packed into tin cups and analyzed by a Thermo Flash EA 1112 elemental analyzer coupled to an IRMS (Thermo Delta Plus XP, Thermo Fisher Scientific). Caffeine was used as a standard for isotope correction and C/N quantification of bulk carbon and nitrogen assimilation. Precisions of the caffeine measurements were: C = 1.07 ± 4.57 × 10^−5^ at% and N = 0.37 ± 6.22 × 10^−5^ at% (*n* = 23).

#### *Extraction of sediment samples and preparation of fatty acid methyl esters*

The bacterial incorporation of added phytodetritus was estimated through the isotope enrichment of bacterial‐specific phospho‐lipid fatty acids (PLFAs) (Boschker and Middelburg 2002). Two main biomarkers were chosen for the analysis, i15:0 and ai15:0, because of their specificity for bacteria and their presence in all samples. Lipid extraction was performed using a modified method from Bligh and Dyer (1959) according to Sturt et al. (2004). In short, lipids were extracted using a mixture of methanol, dichloromethane, and phosphate buffer to pH 7.4 or trichloroacetic acid (2 : 1 : 0.8 v/v). From this total lipid extract, an aliquot (1/2) was saponified using 6% KOH in methanol, after which neutral lipids were released with hexane and subsequently removed (Elvert et al. 2003). The remaining methanolic–water phase was acidified to pH 1, and free fatty acids were extracted with hexane. This extract was dried under a stream of nitrogen, and free fatty acids were reacted with 1 mL 14% BF_3_ in MetOH at 70°C for 1 h to form fatty acid methyl esters (FAMES). FAMES were identified via GC–mass spectrometry (Thermo Quest Trace GC with Trace MS), and concentrations determined by GC–flame ionization detection (Thermo Finnigan Trace GC) relative to the internal standard 2Me‐octadecanoic acid added prior to extraction. Corresponding stable carbon isotope compositions of FAMEs were determined by GC‐IRMS (Thermo Scientific V Delta Plus with Trace GC ultra, connected via GC Isolink and ConFlo IV interfaces) using CO_2_ as a reference and cross checked against the known δ^13^C value of the internal standard FA. δ^13^C values were corrected for the methyl group added during derivatization. δ^13^C values have an analytical error of 1 ‰ based on duplicate injection of selected samples.

#### *Fauna samples*

Frozen sediment samples were washed on a 1 mm and 125 *μ*m sieve. Two depth horizons were considered: the surface (0–1 cm) and subsurface sediment horizon (1–5 cm). Macrofauna (> 1 mm) was sorted under a stereomicroscope in a clean glass vial on ice. The meiofauna > 125 *μ*m to 1 mm was extracted by centrifugation (three cycles) in a colloidal silica solution (Ludox TM‐50) with a density of 1.18 g cm^−3^. Each extraction was again kept in milliQ water in clean glass Petri dishes on ice. Both size fractions of fauna were directly processed on ice to minimize potential leakage of label (Moens et al. 1999).

Organisms were sorted under a stereomicroscope to higher taxon level (meiofauna) or to family, genus, or species level whenever possible (macrofauna). All organisms were cleaned from debris and washed in milliQ water again before they were transferred to small aluminum or silver cups in a drop of milliQ water (meiofauna). About 150 nematodes, 15 ostracods, 15 harpacticoid copepods, and 5 cumaceans, amphipods, isopods, or tanaidaceans were handpicked with a fine sterile needle, rinsed in milliQ water to remove adhering particles, and transferred to a drop of milliQ water in Al, Sn, or Ag cups. Ag cups were used for calcifying organisms, which were acidified with a few drops of 2% (meiofauna) or 10% (macrofauna) HCl. As acidification affects the nitrogen signature of the tissues (Mateo et al. 2008), separate samples were prepared for carbon and nitrogen isotopic signatures of calcifying organisms (all except nematodes, polychaetes, and *A. eightsii* tissue). When insufficient biomass was present in the samples, the samples for ^13^C analysis were prioritized. This resulted in an unbalanced dataset with more observations for ^13^C than for ^15^N. Also, larger macroalgae fragments (> 1 mm, but typically a few mm long) that could be handpicked with tweezers were transferred to tin cups. The Al and Ag cups for meiofauna had been preheated at 500°C to remove any contaminating organic matter that could interfere with the meiofauna stable isotopic signature. The Sn and Ag cups for macrofauna and macroalgae had not been preheated. The cups with the organisms were then oven dried at 60°C until complete dryness, pinch closed, and stored in multiwell plates. Small organisms were encapsulated as a whole, whereas large organisms were grinded and subsampled. The carbon and nitrogen isotopic composition of the macrofauna samples was determined with a PDZ Europa ANCA‐GSL elemental analyzer 230 interfaced to a PDZ Europa 20‐20 IRMS (Sercon Ltd.; UC Davis Stable Isotope Facility). During analysis, samples were interspersed with several replicates of at least two different laboratory standards. These laboratory standards, which are selected to be compositionally similar to the samples being analyzed, have been previously calibrated against NIST Standard Reference Materials (IAEA‐600, USGS‐40, USGS‐41, USGS‐42, USGS‐43, USGS‐61, USGS‐64, and USGS‐65). The long‐term standard deviation is 0.2‰ for ^13^C and 0.3‰ for ^15^N. Because of the higher precision needed for the low biomass meiofauna, these samples were measured on a FlashEA 1112 Elemental Analyzer, coupled to a DeltaV IRMS (both from Thermo Scientific) at NIOZ Yerseke. USGS‐40 (SD ^13^C 0.1‰, SD ^15^N 0.15‰) was used as laboratory standards.

All organisms in the sample were counted to quantify the assimilation in the entire community. Faunal biomass was determined via C content values from the IRMS, combined with faunal abundance.

#### *Calculations*

Rates of total oxygen uptake (TOU), ^13^C‐DIC, ^15^N–NH_4_^+^, ^15^N–NO_x_^−^, and ^15^N‐N_2_ accumulation in the overlying water were calculated from the slope of linear regressions of concentration as a function of time. The fluxes of ^15^N–NH_4_^+^ and ^15^N–NO_x_^−^ at the sediment–water interface are to be considered net fluxes and conservative estimates, as we do not have information on the production and consumption processes in the overlying water or the flux into the sediment. The ^15^N‐N_2_ flux is calculated as the sum of the upward flux (^15^N‐N_2_ release into overlying water) and flux into the sediment (^15^N‐N_2_ release into pore‐water pool). The flux into the sediment was assumed to equal the upward flux multiplied by sediment porosity. The total quantity of ^13^C accumulating in the DIC pool was then estimated by interpolating between measured daily dark respiration and subsequent integration over time. Total DIN fluxes were estimated in the same way.

^13^C accumulation in the bulk sediment (POC, uncharacterized organic carbon), large macroalgae fragments, and macrofauna were calculated as the product of the excess at% ^13^C and the carbon content of the sample, divided by the at% ^13^C of the labeled algae (*see* Eq. (1)). Calculations for ^15^N were made accordingly.

(1) $$C‐\text{accumulation}\;\left(\mu g\;C\right)=\left(\mathrm{at}{\%}^{13}C_{\text{sample}}–\mathrm{at}{\%}^{13}C_{\text{background}}\right)/\mathrm{at}{\%}_{\text{algae}}\times {\mathrm{TOC}}_{\text{sample}}$$

^13^C and ^15^N assimilation in meiofauna biomass was calculated in the same way as for macrofauna, but with excess calculated as indicated in Eqs. 2, 3, 4. Again, calculations for ^15^N were made accordingly.

Excess ^13^C (*E*) is given by the difference in fraction ^13^C in the sample (*F*_sample_) and the background (*F*_background_).

(2) $$E=F_{\text{sample}}–F_{\text{background}}$$

where

(3) $$F\;{=}^{13}C/\left({\;}^{13}C{+}^{12}C\right)=R/\left(R+1\right)$$

and

(4) $$R=\left(\partial^{13}C/1000+1\right)\times R_{\text{VPDB}}$$

where *R*_VPDB_ = 0.0111802 is the carbon isotopic ratio of the Vienna Pee Dee Belemnite standard.

^13^C label incorporation into bacterial biomass was based on the bacterial PLFAs that were present in all incubation chambers and depth horizons (i15:0, ai15:0) (Boschker and Middelburg 2002). For each bacterial PLFA, ^13^C label incorporation was calculated as:

(5) $$I_{\text{PLFA}}=E_{\text{PLFA}}\times {\text{PLFA}}_{\text{carbon concentration}}\;$$

With *E* calculated as in Eqs. 2, 3, 4.

Subsequently, incorporation into bacterial biomass was calculated based on Middelburg et al. (2000) as:

(6) $$I=\mathrm{sum}\;I_{\text{PLFA}}/\left(a\times b\right)$$

where *a* is the average PLFA concentration in bacteria (0.056 g PLFA C g^−1^ of C biomass in sediments (Brinch‐Iversen and King 1990) and *b* is the fraction of the bacterial PLFA considered here that is encountered in sediments of Potter Cove (0.089; calculated from the fraction of i15:0 and ai15:0 in the control sediments, data not shown). ^13^C recycling into microphytobenthos biomass was calculated as for the bacterial assimilation, but using 22:6n3 as a marker for diatom biomass (Volkman et al. 1998), as this PLFA was not present in the added *Desmarestia* and *Palmaria* (data not shown). 20:5n3 or 16:1n7 are usually more abundant PLFAs in microphytobenthos (Volkman et al. 1998), but as these were also important markers for *Desmarestia*, they were not selected as unique markers for microphytobenthos. For benthic diatoms, *a* is the average PLFA concentration in diatom biomass: 0.042 g PLFA C g^−1^ of C biomass in sediments, based on the assumption of 0.035 g PLFA C g^−1^ of C biomass of microphytobenthos grown at ~ 20°C (Volkman et al. 1998), corrected for a 20% increase in PLFA production with a 18°C decrease in temperature (Lynch and Thompson 1982). *b* is the fraction of the diatom PLFA considered here that are encountered in sediments of Potter Cove (0.13; calculated from the fraction of 22:6n3 in the control sediments, data not shown). Only the 0–1‐cm sediment layer was considered for incorporation of ^13^C into microphytobenthos. Bacterial biomass was estimated from the concentration of the unique bacterial markers and the above‐mentioned average PLFA concentration in bacteria (*a*) and the fraction of the bacterial PLFA considered here that are encountered in Potter Cove (*b*). Microphytobenthic biomass was estimated likewise. The total amount of algal C (^12^C + ^13^C) recovered from bacteria, microphytobenthos, meiofauna, and macrofauna and DIC and POC pools were calculated as the quotient of the total accumulation I and the fractional abundance of C in the algae (0.11–0.17). Due to the relatively large differences in fractional abundance of ^13^C and ^15^N in the two macroalgae species, results are presented as macroalgal‐derived C or N, rather than ^13^C or ^15^N. This enables comparison of species specificity in algae assimilation or respiration.

Assimilation into biomass is expressed as total assimilation (summed individual assimilation × density; in mg macroalgal C or N m^−2^) and as carbon‐ or nitrogen‐specific assimilation (total assimilation/biomass in terms of carbon or nitrogen; ng macroalgal C or N organismal *μ*g C^−1^ or N^−1^). The results on carbon‐ and nitrogen‐specific assimilation will not be presented in the results but can be found in a comprehensive form in the Supporting Information Figs. S5–S7.

All data are available at PANGAEA. (doi:10.1594/PANGAEA.897419). Error terms are standard deviation.

## *Results*

The added macroalgal fragments settled at the sediment surface in a few hours’ time. Four days after addition, macroalgal fragments of *Desmarestia* were not visible anymore at the sediment surface, whereas 2 weeks later, *Palmaria* fragments were still discernible. Microbial mats started to develop at the sediment surface of every core after 1 d, although not in every core to the same extent. Macrofauna in the cores was abundant (amphipods, errant polychaetes, burrowing isopods, *A. eightsii* bivalves, cumaceans, and sea pens), and almost all animals were alive at each sampling point. The overlying water in the cores was always clear, except for the *Palmaria* core that was run for 26 d. Even though the overlying water was replaced every 2 d, it always turned turbid again in this core.

### Carbon and nitrogen stocks

The sediment of the study site had an average organic carbon content of 0.21 dry wt% and nitrogen content of 0.04 dry wt% in the upper centimeter. Taking into account a porosity of 0.78 and a sediment density of 2.55 g cm^−3^, this results in a TOC pool of 12 g C_org_ m^−2^ and a TN pool of 2 g N m^−2^ for the upper centimeter. As such, the 2.55 g C_org_ m^−2^ addition of the macroalgae represented an estimated increase of ~ 20% organic carbon in both treatments and 9% (*Desmarestia*) to 14% (*Palmaria*) nitrogen to the background nitrogen content of the surface sediment.

Bacteria made up the largest share of the living biomass in the control sediments (19 ± 3 g C m^−2^; 41–81%), followed by macrofauna (5 ± 5 g C m^−2^; 5–32%), microphytobenthos (4 ± 4 g C m^−2^; 12–31%), and finally meiofauna (0.12 ± 0.15 g C m^−2^; 0.07–1%). Within meiofauna, nematodes were numerically and biomass wise most abundant (Supporting Information Figs. S2, S3). The most abundant macrofauna taxa in the controls were the small bivalve *Mysella* sp., Cumacea, Amphipoda, and Ostracoda. Polychaete worms and the burrowing bivalve *A. eightsii* generally displayed low abundances but were spread vertically over the upper 5 cm of the sediment (Supporting Information Fig. S4). The biomass of the very abundant Cumacea and small Bivalvia like *Mysella* sp. was negligible as compared to *A. eightsii* and Polychaeta (Supporting Information Fig. S5). Similar patterns were observed in the macroalgae treatments.

### TOU and macroalgal DIC release

**Figure 1:**
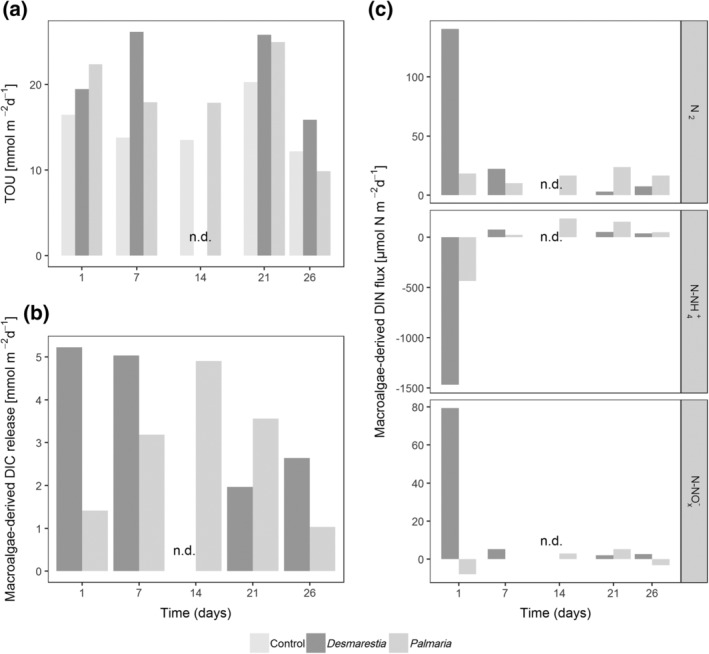
**(a)** TOU, **(b)** macroalgal‐derived DIC release (based on ^13^C‐DIC) and **(c)** macroalgae‐derived fluxes of N_2_, NH_4_^+^‐N, and NO_x_^−^‐N (based on ^15^N‐DIN) in the two macroalgae treatments over time. Positive fluxes are effluxes from the sediment to the overlying water. Negative fluxes represent fluxes into the sediment or consumption at the sediment–water interface.

The average TOU in the control cores was 15.3 ± 3.2 mmol O_2_ m^−2^ d^−1^ over all sampling dates (Fig. 1a). Except for *Palmaria* day 26, TOU was always higher in the macroalgae treatments as compared to the control, resulting in an average of 21.8 ± 5.0 mmol O_2_ m^−2^ d^−1^ in the *Desmarestia* and 18.6 ± 5.7 mmol O_2_ m^−2^ d^−1^ in the *Palmaria* treatments. No consistent time trend could be observed in TOU. However, in all three treatments, the lowest TOU was observed in the 26 d cores.

One day after macroalgae addition, the macroalgae‐derived DIC release rate (calculated from ^13^C‐DIC production and the ^13^C labeling % of the macroalgae) was highest in the *Desmarestia* treatments and declined thereafter (Fig. 1b). The macroalgae‐derived DIC release from the sediment in the *Palmaria* treatments was highest after 2 weeks and declined thereafter. Thus, the DIC release in the *Palmaria* treatments lagged 2 weeks behind that of the *Desmarestia* treatments. Integrated over time (cumulative), the fraction of the added *Desmarestia* carbon that was respired increased from 2% after 1 d to 45% after 26 d. The fraction of added *Palmaria* carbon that was respired also increased from 1% after 1 d to 39% after 26 d (Supporting Information Table S1).

### Macroalgal DIN release

One day after algae addition, a strong macroalgae‐derived ammonium uptake by the sediment (calculated from ^15^N‐NH_4_^+^ consumption and the ^15^N labeling % of the macroalgae) was observed in the *Desmarestia* core (1468 *μ*mol N m^−2^ d^−1^), along with nitrate release (79.38 *μ*mol N m^−2^ d^−1^) (Fig. 1c). Also N_2_ was released from the sediment (78.40 *μ*mol N m^−2^ d^−1^). Seven days after algae addition, there was no further ammonium uptake by the sediment but rather a small release declining over time (from 75.65 after 1 week to 37.23 *μ*mol N m^−2^ d^−1^ after 4 weeks). Also, the release of NO_x_ decreased with time (5.26 *μ*mol N m^−2^ d^−1^ after 1 week to 2.67 *μ*mol N m^−2^ d^−1^ after 4 weeks) similar to the release of N_2_ (13.75 *μ*mol N m^−2^ d^−1^ after 1 week to 4.59 *μ*mol N m^−2^ d^−1^ after 4 weeks). The total macroalgae‐derived DIN mineralization as the sum of the production or consumption of NH_4_^+^, NO_x_^−^, and N_2_ made up 11% of the added *Desmarestia* nitrogen after 1 d but increased to 55% after 26 d.

Note, however, that summing the production or consumption of all measured N species could lead to an overestimation of the total macroalgae‐derived DIN mineralization as this entails counting some N several times. For example, if macroalgal organic N is remineralized to NH_4_^+^, this can be nitrified to NO_x_^−^ and then reduced via denitrification to form N_2_ gas. So in that case, one N would be counted three times.

In the *Palmaria* treatments, the ammonium uptake by the sediment was lower but also peaked on day 1 (437 *μ*mol N m^−2^ d^−1^), accompanied by a smaller nitrate uptake (7.99 *μ*mol N m^−2^ d^−1^) and a small release of N_2_ (11.34 *μ*mol N m^−2^ d^−1^). Seven days later, ammonium started to be released from the sediment, with a maximum after 14 d (186.10 *μ*mol N m^−2^ d^−1^). N_2_ release from the sediment was more gradual over time and with more moderate fluxes (max. 14.81 *μ*mol N m^−2^ d^−1^ after 21 d). The total DIN release accounted for only 2% of the added *Palmaria* nitrogen after 1 d and increased to 18% after 26 d.

### Macroalgae remains in the sediment

**Figure 2:**
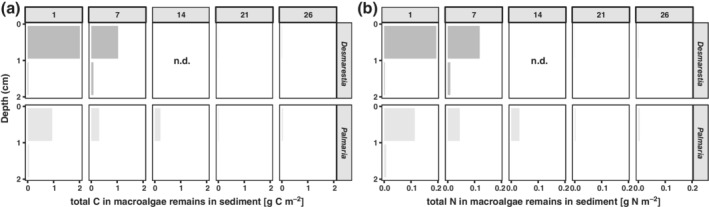
Labeled **(a)** organic carbon and **(b)** nitrogen in large (> 1 mm) *Desmarestia* and *Palmaria* fragments recovered from the upper 2 cm of the sediment (Y axis) in each time step (panels indicate day numbers).

Large macroalgae fragments (> 1 mm) were recovered from the upper 2 cm of the sediment. Mainly *Desmarestia* was recovered, both from the surface and subsurface sediment layer. The biomass of recovered fragments was highest after 1 d (respectively, 80% and 93% of the added *Desmarestia* carbon and nitrogen) and declined thereafter to 44% and 61% of the added *Desmarestia* carbon and nitrogen, respectively, after 7 d (Fig. 2a,b; Supporting Information Table S1). Very few large fragments (< 1% of the added *Desmarestia* carbon and nitrogen) were found in the sediments after 21 and 26 d. The recovery of *Palmaria* fragments was lower (for, respectively, carbon and nitrogen: 39% and 34% after 1 d, 12% and 13% after 7 d, 8% and 9% after 14 d, and < 2% after 21 and 26 d; Supporting Information Table S1), mainly due to the fact that these macroalgae were more easily mechanically disrupted in the sieving and extraction process. A significant amount of the added *Palmaria* ended up as fragments < 1 mm, which were as such not retained for analysis.

### Uncharacterized macroalgae‐derived organic carbon and nitrogen in the sediment

**Figure 3:**
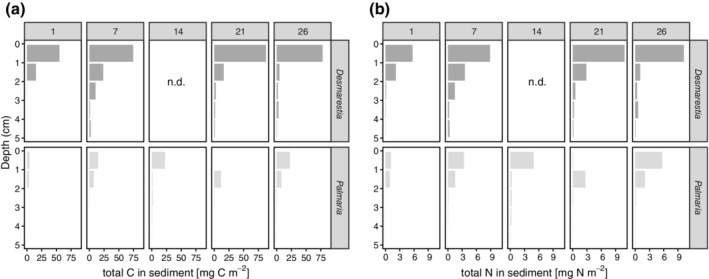
Labeled uncharacterized **(a)** organic carbon and **(b)** nitrogen along the sediment vertical profile of the two macroalgae treatments over time (panels indicate day numbers).

Most of the uncharacterized POC and nitrogen (i.e., bulk POC and PN, possibly containing small macroalgae fragments, bacteria, microphytobenthos, and meiofauna) was found at the surface and decreased with sediment depth (Fig. 3a,b). *Desmarestia* carbon and nitrogen were present in the sediment in higher amounts compared to *Palmaria* carbon and nitrogen. The uncharacterized organic matter of both macroalgae species was also found in the subsurface layers and was at each time step higher in the *Desmarestia* cores (0.15–14.04 mg C m^−2^ and 8.1–14.7 mg N m^−2^) than in the *Palmaria* cores (0.08–5.07 mg C m^−2^ and 2.1–8.0 mg N m^−2^). No clear temporal trend could be observed, and amounts varied little over time. On average, 4% ± 1% of the originally added *Desmarestia* carbon was left as uncharacterized matter in the sediment, compared to only 0.8% ± 0.4% of the originally added *Palmaria* carbon (Supporting Information Table S1). Similarly, on average 6% ± 1% of the *Desmarestia* nitrogen was left as uncharacterized matter in the sediment, compared to only 1.3% ± 0.7% of the *Palmaria* nitrogen.

### Bacterial assimilation

**Figure 4:**
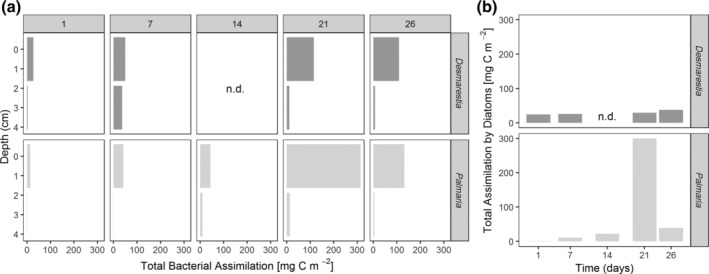
**(a)** Total bacterial assimilation in the surface (0–1 cm) and subsurface (1–5 cm) sediment layers and **(b)** total assimilation by microphytobenthic diatoms in the surface (0–1 cm) sediment layers of the two macroalgae treatments over time (panels indicate day numbers). Bacterial and microphytobenthic assimilation of macroalgal N was not quantified.

Total bacterial assimilation ranged between 0.12 and 314 mg C m^−2^ and increased over time until day 21 (Fig. 4a). In the first week, the assimilation by bacteria was higher for *Desmarestia* carbon than for *Palmaria* carbon, whereas at day 21, a more than two‐fold higher assimilation was observed for *Palmaria* (Fig. 4a; Supporting Information Table S1). Also, a subsurface bacterial assimilation of carbon from the macroalgae was observed and was on all sampling occasions higher in *Desmarestia* cores (ranging between 4 and 37 mg C m^−2^) than in *Palmaria* cores (0.12–12.6 mg C m^−2^), except on the 21st day, where the total assimilation by subsurface bacteria was comparable in both treatments (Fig. 4a). Taken depth layers together, bacteria assimilated 2–6% of the added *Desmarestia* and 1–16% of the added *Palmaria* carbon.

### Recycling by microphytobenthos

Assimilation of macroalgal carbon into microphytobenthic diatom biomass was observed as an increase in labeling of the marker for microphytobenthos (22:6n3), which occurred in both treatments. *Desmarestia* carbon assimilation by diatoms was about 25 mg C m^−2^ in the first week and slightly increased to 37 mg C m^−2^ after 4 weeks (Fig. 4b; Supporting Information Table S1). This represents 1% of the added *Desmarestia* carbon. In the *Palmaria* treatment, carbon recycling by microphytobenthos was lower than that of *Desmarestia*, in the first 2 weeks (1–22 mg C m^−2^) but increased to 300 mg C m^−2^ after 3 weeks (Fig. 4b; Supporting Information Table S1). After 26 d, the carbon assimilation by benthic diatoms in the *Palmaria* core was reduced again. This represents an increase from 0.05% of the added *Palmaria* carbon after 1 d to 12% after 21 d, declining to 2% again after 26 d (Supporting Information Table S1).

### Assimilation by meiofauna

**Figure 5:**
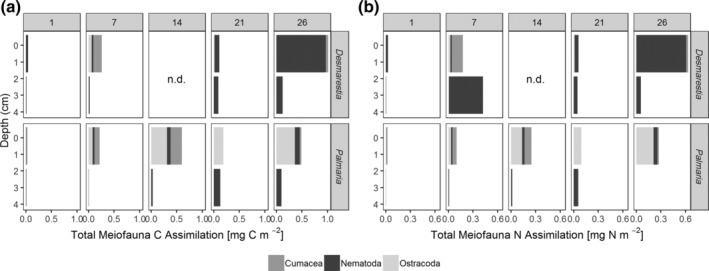
Total assimilation of macroalgae‐derived **(a)** carbon and **(b)** nitrogen by the dominant meiofauna taxa from the surface (0–1 cm) and subsurface (1–5 cm) sediment layers for the two macroalgae species over time (panels indicate day numbers).

Total assimilation of *Desmarestia* carbon and nitrogen by meiofauna increased from 0.1 mg C m^−2^ and 0.06 mg N m^−2^ after 1 d to 2.6 mg C m^−2^ and 1.4 mg N m^−2^ after 26 d (Fig. 5a,b). In the *Palmaria* treatment, assimilation by meiofauna was lower. It increased from 0.04 mg C m^−2^ and 0.02 mg N m^−2^ after 1 d to 0.6 mg C m^−2^ and 0.3 mg N m^−2^ after 14 d, but after 21 and 26 d, the assimilation of *Palmaria* carbon and nitrogen was even lower. The highest total assimilation of carbon and nitrogen occurred in the surface layer, although subsurface nematodes also assimilated macroalgal carbon and nitrogen. Nematoda assimilated the largest shares of *Desmarestia* C and N especially after 3 and 4 weeks, whereas in *Palmaria* treatments, Ostracoda and Cumacea seemed to be the key players throughout the experiment.

In the *Desmarestia* treatment, meiobenthic Tanaidacea and Amphipoda were also important assimilators of macroalgal detritus. These data are not shown as these taxa were not present in the *Palmaria* treatment. If Tanaidacea and Amphipoda are considered as well, this sums up to max. 0.1% of *Desmarestia* carbon and 0.7% of *Desmarestia* nitrogen after 26 d. The meiobenthic assimilation of *Palmaria* was lower: a maximum assimilation of only 0.03% of the added *Palmaria* carbon and 0.08% of the *Palmaria* nitrogen was observed after 14 d (Supporting Information Table S1).

### Assimilation by macrofauna

**Figure 6:**
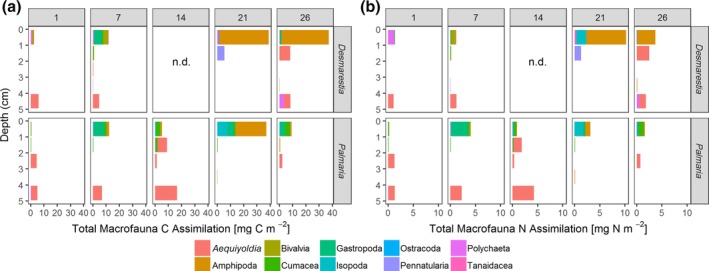
Total assimilation of macroalgae‐derived **(a)** carbon and **(b)** nitrogen by macrofauna for the two macroalgae species over time (panels indicate day numbers) and per sediment layer. Nitrogen is underestimated for calcifying organisms.

Although not dominant in terms of density and biomass, amphipods, the bivalve *A. eightsii*, gastropods and isopods (when present) were the main consumers of the added macroalgae (Fig. 6a,b). Within the amphipods, lysianassoids assimilated high amounts of algal carbon and nitrogen in the *Desmarestia* treatments after 21 and 26 d and in the *Palmaria* treatment after 21 d. In the *Desmarestia* treatments, the total assimilation increased linearly from 9 (1st day) to 54 mg C m^−2^ (26th day), which corresponds to 0.3% to 2% of the added *Desmarestia* carbon being assimilated by macrobenthos (Supporting Information Table S1).

In the *Palmaria* treatments, the total assimilation increased first linearly from 11 (1st day) to 38 mg C m^−2^ (21st day), but then decreased strongly to 12 mg C m^−2^. This corresponds to a range of 0.4 to max. 1.5% of the added *Palmaria* carbon assimilated by macrobenthos (Supporting Information Table S1).

## *Discussion*

We experimentally quantified the degradation pathways of two important macroalgae species—*P. decipiens* and *D. anceps*—from a subtidal area in a subantarctic cove. Increasing glacier melting results in expanding macroalgae growth associated with detritus accumulation at the seafloor in this area. We hypothesized that the degradation of the detritus of the more palatable red alga *P. decipiens* would be faster than that of the less palatable brown alga *D. anceps*.

**Figure 7:**
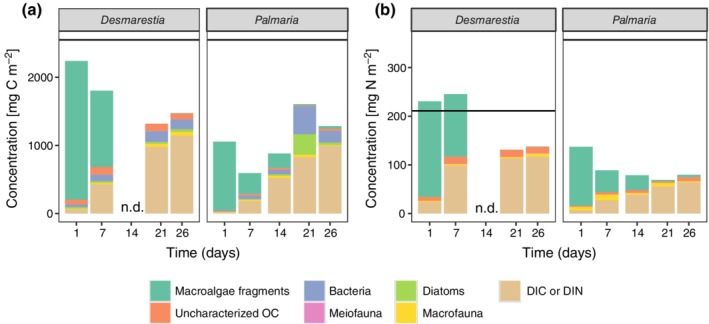
Temporal changes in the concentration of the macroalgal‐derived **(a)** C_org_ and **(b)** N in the different measured compartments of the sediment. The originally added amount of carbon and nitrogen are indicated with a horizontal line. Note that N was not quantified in the bacteria and diatom pool, and N is also underestimated in the macrofauna pool.

The degradation of the macroalgal detritus in this study evolved over time, and the patterns were indeed species specific. After 21 d, 62% of the added *Palmaria* carbon was processed, i.e., respired or assimilated into biomass (here we do not consider the sudden lower amount of processed carbon after 26 d, which we relate to anoxic conditions—see further), as compared to 47% of the originally added *Desmarestia* carbon in 21 d (max. 54% in 26 d; Fig. 7).

From these processed pools (i.e., sum of pools of macroalgae respired and incorporated into bacteria, microphytobenthos, meiofauna, macrofauna except uncharacterized, and macroalgal fragments), we can estimate the time necessary to completely degrade the macroalgae, assuming that decay rates remain the same over time. Linear regressions including all time points except the odd *Palmaria* result at 26 d (*R* ^2^ of linear regressions 0.98 for *Desmarestia* and 0.90 for *Palmaria*) allowed to estimate that the *Desmarestia* detritus would be completely degraded in ~ 7 weeks (48 d), whereas the *Palmaria* detritus would need only ~ 4.5 weeks (31 d).

Nevertheless, our degradation estimates might be overestimated as (1) we studied the degradation of *D. anceps* and *P. decipiens* from the detrital state onward. For logistic reasons, we used young sporophytes, which are less tough and less branched than the adult stages, so probably easier to graze upon by herbivores. In addition, the macroalgae had been freeze dried and shredded, which possibly made them more susceptible to decomposition. In the water column, dead fragments of *D. anceps* degraded faster than fresh fragments (Brouwer 1996). During the decomposition process, the cell walls become more permeable or even disrupt completely, which stimulates leaching and better access for microorganisms to cellular nutrients (Brouwer 1996). (2) The palatability of the algae might have changed during the freezing process: it is unclear whether the unpalatable *D. anceps* cultured under laboratory conditions produces chemical defense metabolites. There are indications that extreme temperatures can degrade the defensive metabolites of fresh macroalgae tissue (Amsler et al. 2012), but short‐term (< 48 h) bioassays showed no amphipod grazing on freeze‐dried and ground alginate pellets of *D. anceps*, suggesting that defensive metabolites were still preventing herbivory to some extent (Huang et al., 2006). However, dead *D. anceps* becomes more palatable only after 2 weeks (Amsler et al. 2012), which agrees with the relatively stronger macrofaunal assimilation after 21 d as observed in this experiment (no data for day 14). (3) Part of the detritus can remain recalcitrant in the sediment, hence might not be decomposed (Krause‐Jensen and Duarte 2016). In case 10% of the macroalgal carbon would remain as recalcitrant fraction in the sediment, the degradation would stop after 27 (*Palmaria*) to 43 d (*Desmarestia*). However, without replication and with the anoxic *Palmaria* core after 26 d displaying lower amounts of processed carbon, our data only suggest a faster recycling of *Palmaria* vs. *Desmarestia*, so the first hypothesis is not fully supported. The lack of replication is a potential drawback of our study. The high variability between cores in carbon stocks among compartments (Supporting Information Fig. S5a) suggests that many replicates would have been needed to test for significant differences between the two algal treatments. Collecting and incubating replicate cores at each of the five sampling times was logistically not feasible. However, trends between time intervals were in many cases consistent, supporting the reliability of the observations. The consistent increase in assimilation of carbon and nitrogen into biota over time, along with the decrease in macroalgal fragments found in the sediment shows that the processes taking place are generally not an artifact caused by (spatial) variation between cores but reflect real changes over time.

In addition, we observed clear differences in the degradation pathways between the two species of macroalgae: the early degradation of *Desmarestia* was more microbial driven, whereas the degradation of *Palmaria* was more herbivore driven. Respiration of *Desmarestia* started immediately after addition, as seen in the highest macroalgal‐derived DIC release 1 d after the addition, and decline thereafter (Figs. 1b, 7). The immediate high DIC release in the *Desmarestia* treatment was accompanied by a strong ammonium uptake. This high ammonium uptake must have been preceded by a strong release of ammonium directly after algae addition on day 0 (hence not quantified). Accumulation of dissolved substances is probably a result of leaking of the shredded *Desmarestia* detritus. The abundant ammonium was quickly nitrified (as seen in the strong ^15^N‐NO_x_^−^ release at day 1) and further converted into N_2_, a result of either denitrification or anammox. At the end of the incubation at day 1, the ^15^N‐NH_4_^+^ concentration was 8 *μ*mol L^–1^, while there was only 0.13 *μ*mol L^–1 15^N‐NH_4_^+^ present in the overlying water at the start of the incubation at day 7. The decrease of 4 *μ*mol L^–1 15^N‐NH_4_^+^ per day measured at day 1 implies that the ^15^N‐NH_4_^+^ concentration had declined to normal concentrations at day 3. Hence, from day 7 onward, the measured DIN fluxes are likely to be more representative of actual *Desmarestia* degradation.

In contrast, the maximum DIC release in the *Palmaria* treatments had a time‐lag of 2 weeks with respect to the *Desmarestia* treatments (Figs. 1b, 7). The observed time lag of 2 weeks in the maximum release of *Palmaria‐*derived DIC, together with small *Palmaria*‐derived nitrogen fluxes, suggests a delay in bacterial degradation, which would agree with an initially stronger role for macrofauna in the first 2 weeks of *Palmaria* detritus recycling (macrofauna assimilation decreases from 23% of the processed *Palmaria* carbon pool after 1 d to 1% after 26 d). Macrofauna assimilation was also initially more similar to bacterial assimilation (ratio bacterial over macrofauna assimilation 2–3), whereas bacterial assimilation dominated over macrofaunal assimilation in the later stages of the degradation of *Palmaria* (ratio bacterial over macrofauna assimilation 11–14). As *Palmaria* is a more palatable species for herbivores (Amsler et al. 2005), the macrofauna at the surface could have immediately ingested the added *Palmaria* detritus. Macrofauna has a competitive advantage over bacteria when food is patchily distributed such as in large macroalgae chunks, whereas bacteria are more favored when food is distributed homogeneously (van Nugteren et al. 2009). The latter may have been the case in the later stages of the degradation, as also evidenced by the lack of large *Palmaria* remains after 21 and 26 d. Also, Kristensen et al. (1992) observed competitive interaction for the labile parts of macroalgae between polychaetes and heterotrophic bacteria. It seems that macrofauna had a comparatively stronger role in the first stages of the degradation of *Palmaria*, whereas bacteria dominated the later stages when the food was more diluted in the sediment. *Desmarestia,* on the other hand, is known as less palatable to herbivores (Amsler et al. 2005), so we expected lower grazing of macrofauna on *Desmarestia* carbon than on *Palmaria* carbon. In terms of total assimilation, this was not the case. However, the amount of large *Desmarestia* remains still present in the sediment in the first week was remarkably higher as compared to the amount of *Palmaria* fragments in that first week. This may suggest that macrofauna assimilated the *Desmarestia*‐derived carbon indirectly, by grazing more on bacteria and microphytobenthos that had incorporated the macroalgal carbon. The maximum assimilation of *Desmarestia* carbon by macrofauna came with a delay of at least 14 d (Figs. 6a, 7), which corresponds with the time frame in which the palatability of *D. anceps* increases (Amsler et al. 2012). Bacterial growth probably made the *Desmarestia* fragments more palatable, as suggested by the higher bacterial assimilation of *Desmarestia* after 1 and 7 d as compared to *Palmaria*. In addition, the *Desmarestia* organic matter was buried more in the subsurface layers than that of *Palmaria*, probably because it was disregarded by surface herbivores. This burial by deep burrowing bivalves such as *A. eightsii* and upward conveying Maldanid polychaetes enables subsurface bacteria to incorporate the *Desmarestia* carbon and nitrogen, as seen in higher total and carbon‐specific assimilation (total assimilation standardized for carbon biomass) by bacteria in the subsurface sediment layers of the *Desmarestia* cores (Supporting Information Fig. S6).

### Differences between food web compartments

The bacteria in the present study responded within 1 d to the addition of macroalgal detritus, and the bacterial biomarkers also showed a consistent enrichment of ^13^C throughout the 26 d. This is consistent with microbial‐mediated degradation of macroalgae (Hardison et al. 2010). Heterotrophic bacteria probably assimilated the DOM leaching from the macroalgae into their biomass and they mineralized the remainder into the pore water as DIC and DIN. Once the macroalgal biomass is hydrolyzed to DOM, it is efficiently exchanged between bacteria and microphytobenthos in organic and inorganic forms. This is supported by the clear assimilation of macroalgal carbon in the marker for microphytobenthos. Also, the decline in large macroalgae fragments from day 14 onward, together with fluxes of ^15^N‐DIN shows that while the macroalgae biomass has disappeared, the dissolved substances from the detritus are shuffled between the different compartments of the food web. This efficient recycling of labile carbon has been observed in several other studies across latitudes (Middelburg et al. 2000; Hardison et al. 2010; Oakes and Eyre 2014), including the Arctic (Oakes et al. 2016). Trophic interactions between microphytobenthos and mobile grazers within the macrofauna and meiofauna size classes might then accelerate the recycling of detritus (Rossi 2007). In the end, these interactions between primary and secondary decomposers and grazers of different trophic levels result in a longer retention of macroalgal detrital products in the sediment (Hardison et al. 2010; Rossi et al. 2013). These observations suggest that the sediment in Potter Cove retains the nitrogen and carbon released from the macroalgae degradation process, whereas a relatively small amount of macroalgal nutrients is released back into the overlying water, with a negative feedback on phytoplankton production in the water column. This “sink effect” would also explain the prolonged food availability for the benthos (so called “food bank”; McClintic et al. 2008; Mincks et al. 2005) in the euphotic Antarctic sediments in the form of microphytobenthic biomass, at least as long as the overlying waters are not too turbid or ice covered.

Meiobenthos is thought to be trophically linked to detritus and microorganisms (Coull 1999; Moens et al. 2014). Macroalgae in this experiment were probably indirectly consumed by bacterivorous nematodes feeding on the bacterial communities developing on the fragmented algal detritus (Alkemade and Van Rijswijk 1993; Urban‐Malinga and Burska 2009) or by nonselective deposit feeders and epistrate feeders grazing on microphytobenthos that was benefiting from the dissolved substrates released by the macroalgal detritus. This would agree with observations in an earlier selectivity experiment, where nonselective deposit feeders and epistrate feeders were the nematode groups that preferred *P. decipiens* fragments over other food sources (Pasotti 2015). The similar carbon‐ and nitrogen‐specific assimilation (i.e., total assimilation standardized for carbon or nitrogen biomass, respectively; data presented in detail in Supporting Information Fig. S7) of *Desmarestia* and *Palmaria* indeed suggests nonselectivity or indirect grazing by nematodes and cumaceans. Ostracoda, on the other hand, displayed a higher carbon‐ and nitrogen‐specific assimilation of *Palmaria* carbon and nitrogen compared to that of *Desmarestia* (Supporting Information Fig. S7). There are different mechanisms by which ostracods can assimilate macroalgal organic carbon. Benthic ostracods are known to be able to filter feed or graze detritus and feed on microphytobenthos (Montagna et al. 1995; Buffan‐Dubau and Carman 2000), but some species also graze on exudates of littoral seaweeds (Williams 1969). The taxon specificity in the preference for the added macroalgae observed during this study suggests also direct grazing on *Palmaria* detritus by the present ostracod species.

Planktonic diatoms, benthic diatoms, or bacteria seem to be all potential food sources for meiofauna in Potter Cove both in trophic studies and morphological observations (Pasotti et al., 2012, 2015b,*b*; F. Pasotti unpubl.). Meiofauna organisms are usually dominated in numbers (and in this study also in terms of biomass) by nematodes, which are capable of different feeding strategies. In Potter Cove, the dominant feeding strategies among the nematodes assemblages are epistrate feeders (feeding on diatoms) and deposit feeders (feeding mostly on bacteria; Pasotti et al. 2014). In light of the lower biomass that nematodes represented compared to that of macrofauna and the bacteria (up to four orders of magnitude smaller), the very low contribution of meiofauna (max. 1% of the added macroalgal biomass) to the degradation of the given labeled organic matter is comparable to what is measured in other studies applying stable isotopes in the shallow Antarctic (Moens et al. 2007; Pasotti et al. 2012). The meiofauna carbon‐specific assimilation of macroalgal carbon matched the lowest macrofaunal carbon‐specific assimilation (e.g., *A. eightsii* and ostracods; compare Supporting Information Figs. S7, S8), showing that, even when taking into account their low total biomass, the meiofaunal organisms assimilate only small amounts of added carbon, as has been repeatedly observed in such enrichment experiments (e.g., Middelburg et al. 2000; Braeckman et al. 2011; Pasotti et al. 2012). An often used explanation for these lower assimilation rates is that meiofauna graze nonselectively on unlabeled, older, organic matter within the sediment.

Amphipods, gastropods, the bivalve *A. eightsii*, and isopods (when present) were the main consumers of the macroalgal detritus within the macrofauna size class. A high assimilation of macroalgal detritus by intermediate predators such as amphipods, isopods, and gastropods was suggested in an earlier food web model study of Potter Cove, based on their low mean trophic level (Marina et al. 2018). The observed assimilation of macroalgal carbon and nitrogen could have been achieved either directly or indirectly through grazing on bacteria and microphytobenthos that had already recycled the macroalgal DOM. Early studies indeed report grazing of intertidal amphipods on partly degraded thalli from red and brown algae (Reichardt and Dieckmann 1985). We observed remarkably high carbon‐ and nitrogen‐specific assimilation by lysianassoid amphipods and gastropods (Supporting Information Fig. S8). Lysianassoid amphipods are opportunistic scavengers, primarily feeding on nototheniid fish carcasses (Slattery and Oliver 1986). One species, *Cheirimedon femoratus,* has become the numerically dominant (> 99%) lysianassoid amphipod species in the newly ice‐free areas of inner Potter Cove, where nototheniid fish carcasses are scarce (Seefeldt et al. 2017b). As such, *C. femoratus* has been forced to make a dietary switch, grazing on a vegetarian diet of *P. decipiens* and *D. menziesii* instead (Lastra et al. 2014; Seefeldt et al. 2017a). As our samples originate from inner Potter Cove, the lysianassoid amphipods in this study may most likely be *C. femoratus*, which would explain the high assimilation of macroalgal detritus. Also, gastropods are known herbivores of some macroalgae and their epiphytes. As an example, *Laevilacunaria antarctica* is a herbivorous gastropod not affected by the chemical deterrents produced by brown algae (Iken 1999). The assimilation of algal detritus by gastropods just 1 week after addition of *Palmaria* and *Desmarestia* detritus, both in terms of total and specific carbon assimilation (Supporting Information Fig. S8), would confirm this feeding strategy for the species found within our cores.

The actively burrowing bivalve *A. eightsii* was the first major contributor to the total macrofauna assimilation of the algal detritus, partly due to its high individual biomass (large individuals). *A. eightsii* is a detritus feeder, living most of the time buried 2–3 cm deep in the sediment (Davenport 1988). This bivalve takes sediment into its mantel cavity and mixes it with mucus. During the sorting process, most of the sediment‐mucus slug is expelled as pseudofeces and only 0.6% is ingested as food (Davenport 1988). This intense reworking of the sediment and mixing with mucus probably results in a very attractive substrate for microbial communities, which must have contributed to the bacterial degradation of the added macroalgal detritus. Previous studies have emphasized the influence of brown algal carbon in food webs along the western Antarctic Peninsula, primarily through detritus consumed by the bivalve *A. eightsii* among other deposit feeders (Dunton 2001). Along with the upward conveying polychaetes belonging to the Maldanidae, *A. eightsii* might have contributed to the burial of the macroalgal detritus into the sediment. Burial and even “gardening” of macroalgae by polychaetes and bivalves is well‐documented for shallow coastal sediments (Hylleberg 1975; Woodin 1977; Levin et al. 1997). The sediment reworking activities of polychaetes have been found to increase the cycling of macroalgal carbon and nitrogen (Kristensen et al. 1992; Hansen and Kristensen 1998), but the burial could as well make the fresh organic carbon unavailable to surface feeders and result in subsurface hypoxic conditions (Rossi et al. 2013). High detrital load and lack of oxygen can slow down mineralization processes (Kristensen and Hansen 1995), which is perhaps what we observed in the *Palmaria* core that was incubated for 26 d. In contrast with all other cores, this *Palmaria* core had consistently turbid overlying water throughout the experiment and showed black spots in the sediment. This suggests that a rather large organism was damaged during core sampling and started decaying. This then resulted in hypoxic conditions, hence the observed lower assimilation into biomass and respiration rate as compared to earlier observations.

### Budget

**Figure 8:**
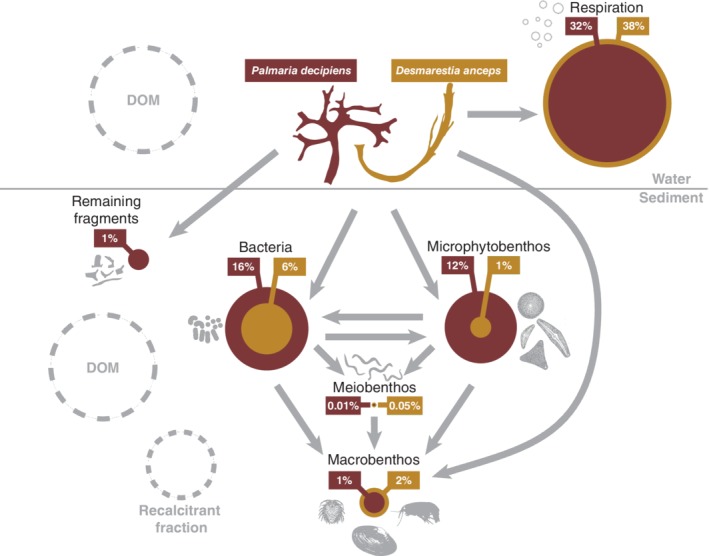
Conceptual figure displaying the percentage of macroalgal carbon that ended up in the different compartments of the benthic ecosystem after 21 d. Assuming that 0–10% of the detritus is recalcitrant (Nielsen et al. 2004), the fraction remaining in the interstitial or overlying water as DOM would represent 36–46% (*Desmarestia*) and 28–38% (*Palmaria*). As these two fractions have not been measured, they are displayed as dashed gray circles. Uncharacterized POC and PN are not shown here as these fractions could be redundant with characterized fractions.

The macroalgae mineralization pattern in this study categorizes our site as one with active faunal uptake (reviewed by Woulds et al. [2009]). Such sites are commonly found in shallow ecosystems with high surface productivity, plenty of food available to the benthos, and consequently large faunal biomass. In these systems, respiration typically accounts for < 75% of the processed carbon (here: on average 67%) and faunal assimilation ranges between 10% and 25% (here, on average 6%) of the processed carbon. Nevertheless, the bacterial uptake in our experiment was also substantial, comprising on average 20% of the processed carbon. After 3–4 weeks, 54% (*Desmarestia*) and 62% (*Palmaria*) of the added macroalgal detritus was traced as being respired, contained in bacterial biomass, microalgae, meiofauna, or macrofauna. Assuming that 0–10% of the detritus is recalcitrant (Nielsen et al. 2004), the fraction remaining in the interstitial or overlying water as DOM would represent 36–46% (*Desmarestia*) and 28–38% (*Palmaria*) (Fig. 8).

The carbon and nitrogen budgets could not be completely closed. This discrepancy can be related to several aspects: (1) only large macroalgal remains were recovered from the sediments, while they may have been partly transformed into very small pieces that were not retrievable anymore, hence not quantified; (2) the DIC and DIN pore‐water pool, the DOC and DON pore water, and the overlying water pool were not quantified in this study. Some carbon and nitrogen may have been converted to DOM, accumulating in the pore water and overlying water, whereas DIC and DIN may have accumulated in in the pore water; (3) the recalcitrant fraction of the macroalgae was not quantified. These unmeasured pools may explain the difference between the amount of macroalgal carbon and nitrogen added and the quantified pool. For nitrogen, the assimilation into bacteria, diatoms, and calcifying macrofauna organisms is also missing. Finally, (4) the unclosed carbon and nitrogen budgets might be a result of accumulated errors in integration procedures due to spatial variability or uncertainties in conversion factors (Middelburg et al. 2000). For these reasons, the nature of the missing carbon and nitrogen cannot be fully resolved.

## *Conclusions*

The carbon from the palatable red alga *P. decipiens* detritus was faster recycled than that of the less palatable *D. anceps*, with herbivores such as amphipods possibly playing a stronger role in the early degradation process of the *Palmaria* fragments and the microbial biota taking over at a later stage. In contrast, *Desmarestia* was more buried in the subsurface sediments, stimulating subsurface bacterial degradation. Macrofauna most likely grazed more indirectly on *Desmarestia* carbon recycled by bacteria and microphytobenthos. The efficient multileveled cycling of the nutrients and carbon from the macroalgae supports a positive feedback loop among bacteria, microphytobenthos, meiofaunal, and macrofaunal consumers, resulting in longer term retention of macroalgal nutrients in the sediment and hence creates a food bank for the benthos. To comprehensively understand the importance of the macroalgae biomass for the benthic ecosystem of Potter Cove, the fraction of macroalgae production ending up in the sediment should be estimated in future studies. This would enable a revision of the budget of macroalgal production, decomposition, and export in Potter Cove (Quartino et al. 2008). As macroalgae are the dominant species covering most of the rocky bottoms of many coastal areas along the western Antarctic Peninsula, the expansion of these key components in other western Antarctic Peninsula locations as a consequence of glacier retreat and other climate change‐related effects may be expected in the near future. The accumulation of the resulting detritus coming from these dominant primary producers on Antarctic subtidal sediments might increase the organic load of the sediment, which, in concert with warming, may lead to higher benthic mineralization rates and finally locally oxygen‐depleted soft sediments. When recalcitrant fractions become buried in such anoxic subsurface sediments, they will significantly contribute to carbon storage and provide a negative feedback on atmospheric CO_2_ levels.

## Conflict of Interest

None declared.

## Supporting information

Figure S1: *Desmarestia* and *Palmaria* detrital fragments as added to the sediments in the cores. Fragments recovered from the cores one day after addition.
Figure S2: Meiofauna densities in the controls, *Desmarestia* and *Palmaria* treatments in the surface (0‐1cm) and subsurface layer (1‐5 cm) over the time course of the experiment (panels indicate day numbers). (a) total meiofauna densities, (b) excluding Nematoda.
Figure S3: Meiofauna biomass in terms of (a) carbon and (b) nitrogen in the controls, *Desmarestia* and *Palmaria* treatments in the surface (0‐1cm) and subsurface layer (1‐5 cm) over the time course of the experiment (panels indicate day numbers).
Figure S4: Macrofauna densities in the controls, *Desmarestia* and *Palmaria* treatments in the five sediment horizons over the time course of the experiment (panels indicate day numbers).
Figure S5: Macrofauna biomass in terms of (a) carbon and (b) nitrogen in the *Desmarestia* and *Palmaria* treatments in the five sediment horizons over the time course of the experiment (panels indicate day numbers).
Figure S6: Carbon‐specific assimilation by bacteria in the macroalgae treatments over time and in the surface (0‐1 cm) and subsurface (1‐5 cm) sediment layers.
Figure S7: Specific assimilation of (a) carbon and (b) nitrogen by the dominant meiofauna taxa in the macroalgae treatments over time and in the surface (0‐1 cm) and subsurface (1‐5 cm) sediment layers.
Figure S8: Specific assimilation of (a) carbon and (b) nitrogen by macrofauna in the macroalgae treatments over time. Plots are ordered according to increasing maximum assimilation.
