# Supplementary material for: Degradation of macroalgal detritus in shallow coastal Antarctic sediments
Source: Limnol Oceanogr. 2019 Feb 5;64(4):1423–41. doi: 10.1002/lno.11125 (PMC6774326; doi:10.1002/lno.11125)
Supplement: Supplementary file 1 — Figure S1: Desmarestia and Palmaria detrital fragments as added to the sediments in the cores. Fragments recovered from the cores one day after addition. Figure S2: Meiofauna densities in the controls, Desmarestia and Palmaria treatments in the surface (0‐1cm) and subsurface layer (1‐5 cm) over the time course of the experiment (panels indicate day numbers). (a) total meiofauna densities, (b) excluding Nematoda. Figure S3: Meiofauna biomass in terms of (a) carbon and (b) nitrogen in the controls, Desmarestia and Palmaria treatments in the surface (0‐1cm) and subsurface layer (1‐5 cm) over the time course of the experiment (panels indicate day numbers). Figure S4: Macrofauna densities in the controls, Desmarestia and Palmaria treatments in the five sediment horizons over the time course of the experiment (panels indicate day numbers). Figure S5: Macrofauna biomass in terms of (a) carbon and (b) nitrogen in the Desmarestia and Palmaria treatments in the five sediment horizons over the time course of the experiment (panels indicate day numbers). Figure S6: Carbon‐specific assimilation by bacteria in the macroalgae treatments over time and in the surface (0‐1 cm) and subsurface (1‐5 cm) sediment layers. Figure S7: Specific assimilation of (a) carbon and (b) nitrogen by the dominant meiofauna taxa in the macroalgae treatments over time and in the surface (0‐1 cm) and subsurface (1‐5 cm) sediment layers. Figure S8: Specific assimilation of (a) carbon and (b) nitrogen by macrofauna in the macroalgae treatments over time. Plots are ordered according to increasing maximum assimilation. [file LNO-64-1423-s001.pdf]

Supplementary material to

Braeckman et al. (2019). Degradation of macroalgal detritus in shallow coastal Antarctic sediments. Limnology and Oceanography.

Table S1: Macroalgal-derived carbon in the different compartments in mg C m-2, both in processed pools (assimilation into biomass and respiration) and total quantified pools (processed + uncharacterized Corg + large macroalgal fragments). Values between brackets represent percentages of processed pool (regular font), quantified pool (bold) and total added carbon (bold, italic).

		**Treatment**	***Desmarestia***					***Palmaria***				
		**Time (days)**	1	7	14	21	26	1	7	14	21	26
**Quantified**	Processed	*Bacteria*	38.4	96.7	NA	156.1	144.7	18.2	53.2	64.2	411.1	170.6
			(29%)	(17%)		(13%)	(10%)	(39%)	(20%)	(10%)	(26%)	(14%)
			**(2%)**	**(5%)**		**(12%)**	**(10%)**	**(2%)**	**(9%)**	**(7%)**	**(26%)**	**(13%)**
			(2%)	***(4%)***		***(6%)***	***(6%)***	***(1%)***	***(2%)***	***(3%)***	***(16%)***	***(7%)***
		Diatoms	23.9	25.4	NA	28.8	37.2	1.2	10.6	22.1	299.9	39.2
			(18%)	(4%)		(2%)	(3%)	(3%)	(4%)	(3%)	(19%)	(3%)
			**(1%)**	**(1%)**		**(2%)**	**(3%)**	**(0.1%)**	**(2%)**	**(3%)**	**(19%)**	**(3%)**
			(1%)	***(1%)***		***(1%)***	***(1%)***	***(0.05%)***	***(0.4%)***	***(1%)***	***(12%)***	***(2%)***
		Meiofauna	0.1	0.3	NA	1.4	2.6	0.0	0.2	0.6	0.3	0.6
			(0.06%)	(0.05%)		(0.12%)	(0.19%)	(0.08%)	(0.08%)	(0.10%)	(0.02%)	(0.05%)
			**(0.00%)**	**(0.02%)**		**(0.11%)**	**(0.18%)**	**(0.00%)**	**(0.04%)**	**(0.07%)**	**(0.02%)**	**(0.05%)**
			(0.00%)	***(0.01%)***		***(0.05%)***	***(0.10%)***	***(0.00%)***	***(0.01%)***	***(0.03%)***	***(0.01%)***	***(0.02%)***
		Macrofauna	8.6	18.2	NA	44.4	54.0	10.6	19.4	32.1	37.9	11.8
			(6%)	(3%)		(4%)	(4%)	(23%)	(7%)	(5%)	(2%)	(1%)
			**(0.4%)**	**(1%)**		**(3%)**	**(4%)**	**(1%)**	**(3%)**	**(4%)**	**(2%)**	**(1%)**
			(0.3%)	***(1%)***		***(2%)***	***(2%)***	***(0.4%)***	***(1%)***	***(1%)***	***(1%)***	***(0.5%)***

		Respiration	62.7	432.2		978.2	1144.1	16.9	182.5	522.3	827.0	992.4
			(47%)	(75%)		(81%)	(83%)	(36%)	(69%)	(81%)	(52%)	(82%)
			**(3%)**	**(24%)**		**(74%)**	**(78%)**	**(2%)**	**(31%)**	**(59%)**	**(52%)**	**(77%)**
			(2%)	***(17%)***		***(38%)***	***(45%)***	***(1%)***	***(7%)***	***(20%)***	***(32%)***	***(39%)***
	Unprocessed	*Uncharacterized* C_{org}	70.6	111.8	NA	109.1	88.6	7.5	22.3	26.2	12.4	31.5
			**(3%)**	**(6%)**		**(8%)**	**(6%)**	**(1%)**	**(4%)**	**(3%)**	**(1%)**	**(3%)**
			(3%)	***(4%)***		***(4%)***	***(3%)***	***(0.3%)***	***(1%)***	***(1%)***	***(0.5%)***	***(1%)***
		Macroalgae fragments	2035.5	1118.7	NA	0.1	4.4	1001.0	305.7	214.6	17.0	37.2
			**(91%)**	**(62%)**		**(0%)**	**(0.3%)**	**(95%)**	**(52%)**	**(24%)**	**(1%)**	**(3%)**
			(80%)	***(44%)***		***(0%)***	***(0.2%)***	***(39%)***	***(12%)***	***(8%)***	***(1%)***	***(1%)***
		Total processed	133.7	572.8	NA	1208.9	1382.6	47.0	265.9	641.4	1576.3	1214.7
			(5%)	***(22%)***	***NA***	***(47%)***	***(54%)***	***(2%)***	***(10%)***	***(25%)***	***(62%)***	***(48%)***
		**Total quantified**	2239.9	1803.3	NA	1318.1	1475.6	1055.5	593.8	882.2	1605.7	1283.4
			(88%)	***(71%)***	***NA***	***(52%)***	***(58%)***	***(41%)***	***(23%)***	***(35%)***	***(63%)***	***(50%)***
		Total added	***2550***									

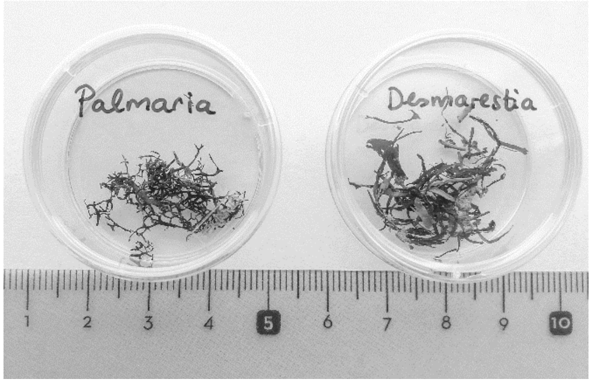

Figure S1: *Desmarestia* and *Palmaria* detrital fragments as added to the sediments in the cores. Fragments recovered from the cores one day after addition.

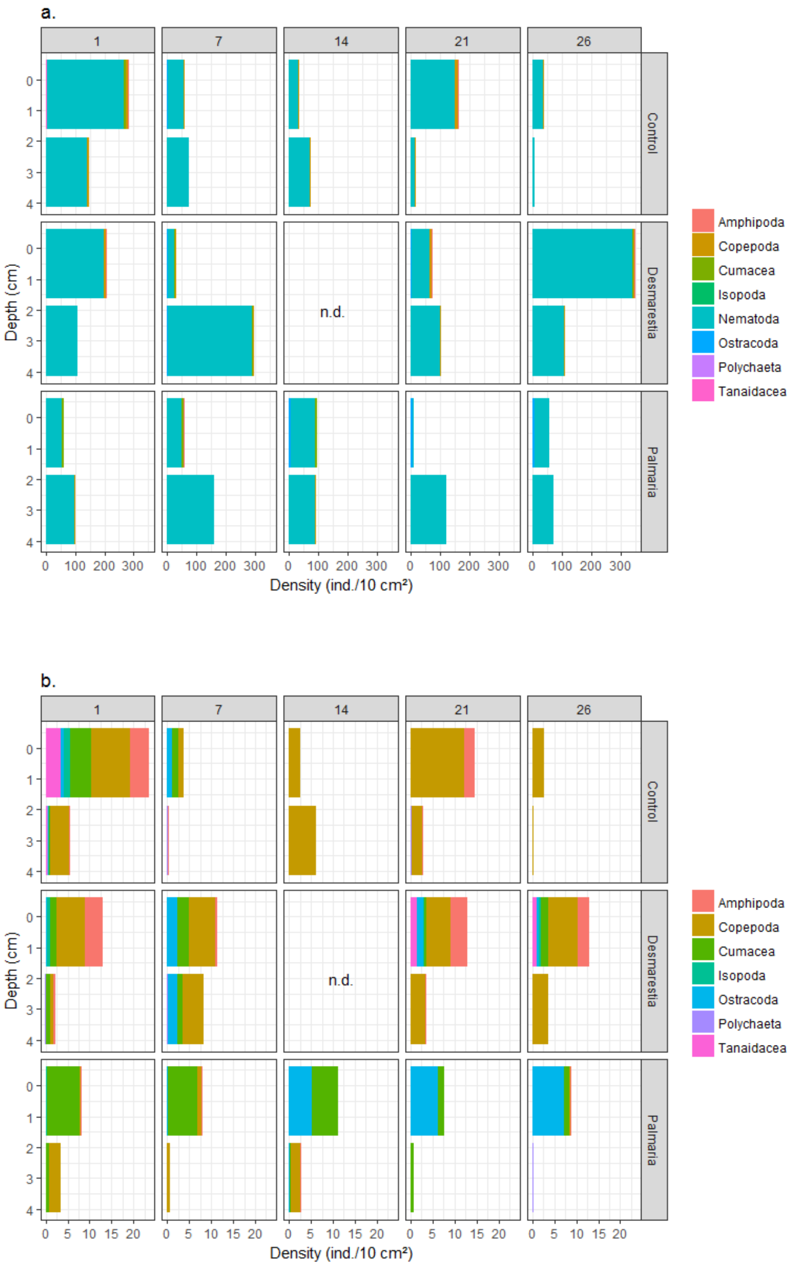

Figure S2: Meiofauna densities in the controls, *Desmarestia* and *Palmaria* treatments in the surface (0-1cm) and subsurface layer (1-5 cm) over the time course of the experiment (panels indicate day numbers). (a) total meiofauna densities, (b) excluding Nematoda.

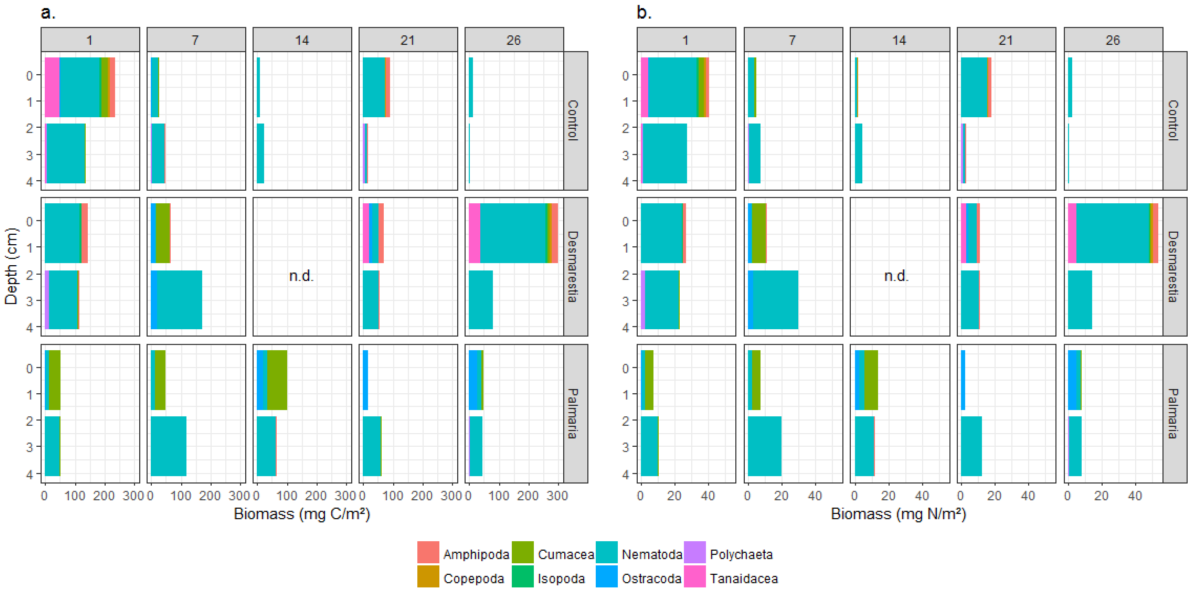

Figure S3: Meiofauna biomass in terms of (a) carbon and (b) nitrogen in the controls, *Desmarestia* and *Palmaria* treatments in the surface (0-1cm) and subsurface layer (1-5 cm) over the time course of the experiment (panels indicate day numbers).

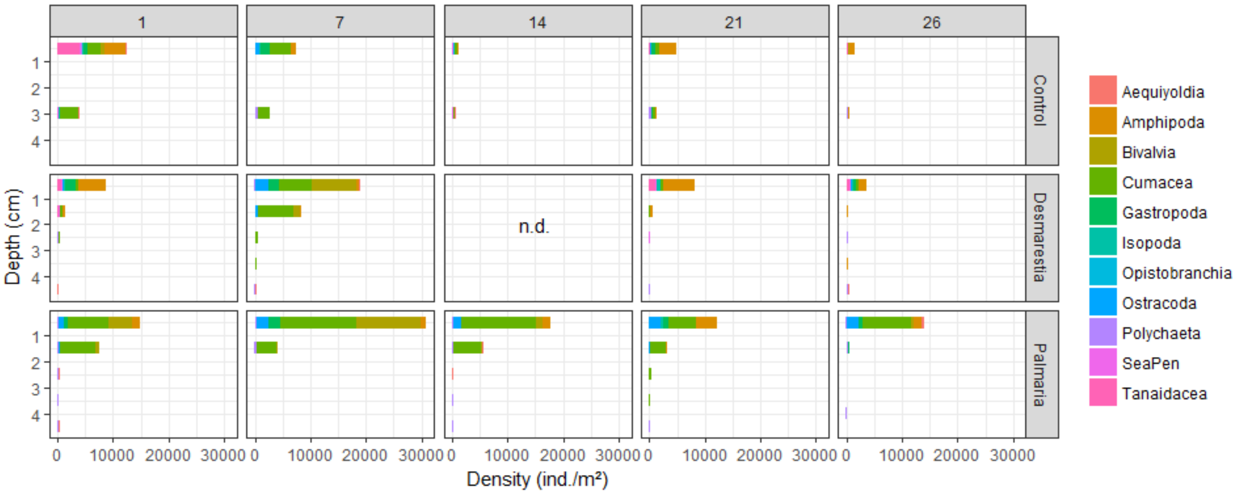

Figure S4: Macrofauna densities in the controls, *Desmarestia* and *Palmaria* treatments in the five sediment horizons over the time course of the experiment (panels indicate day numbers).

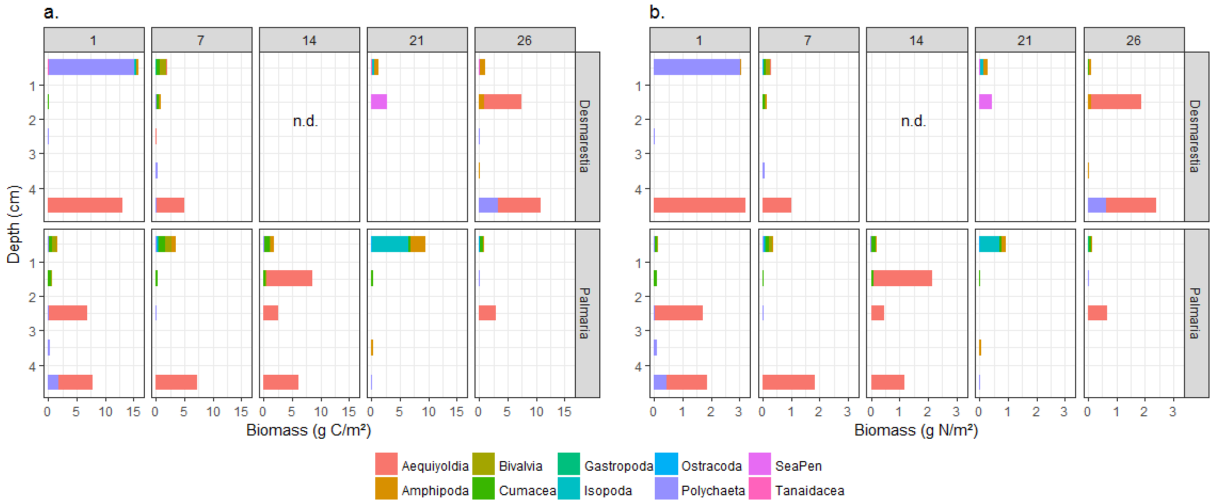

Figure S5: Macrofauna biomass in terms of (a) carbon and (b) nitrogen in the *Desmarestia* and *Palmaria* treatments in the five sediment horizons over the time course of the experiment (panels indicate day numbers).

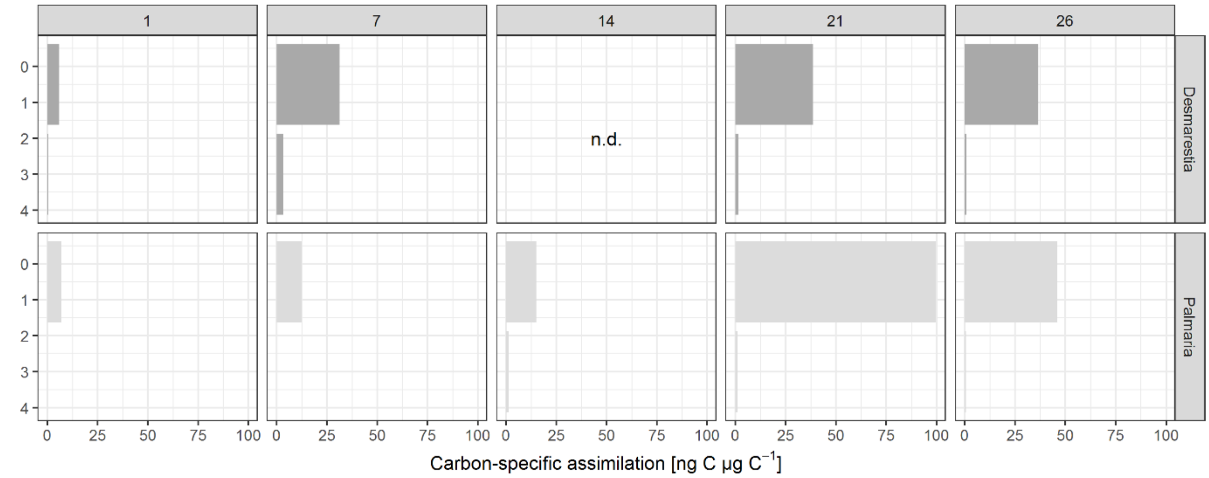

Figure S6: Carbon-specific assimilation by bacteria in the macroalgae treatments over time and in the surface (0-1 cm) and subsurface (1-5 cm) sediment layers.

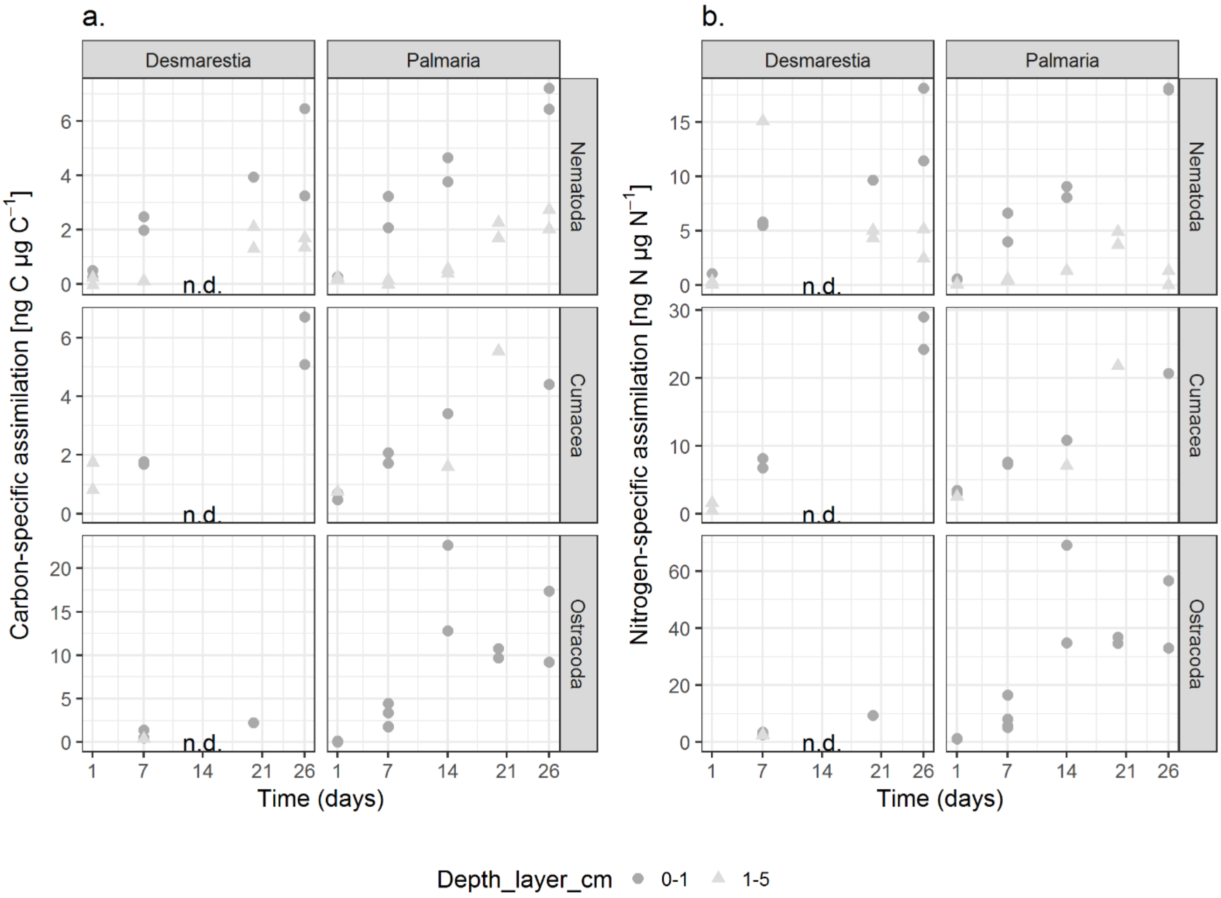

Figure S7: Specific assimilation of (a) carbon and (b) nitrogen by the dominant meiofauna taxa in the macroalgae treatments over time and in the surface (0-1 cm) and subsurface (1-5 cm) sediment layers.

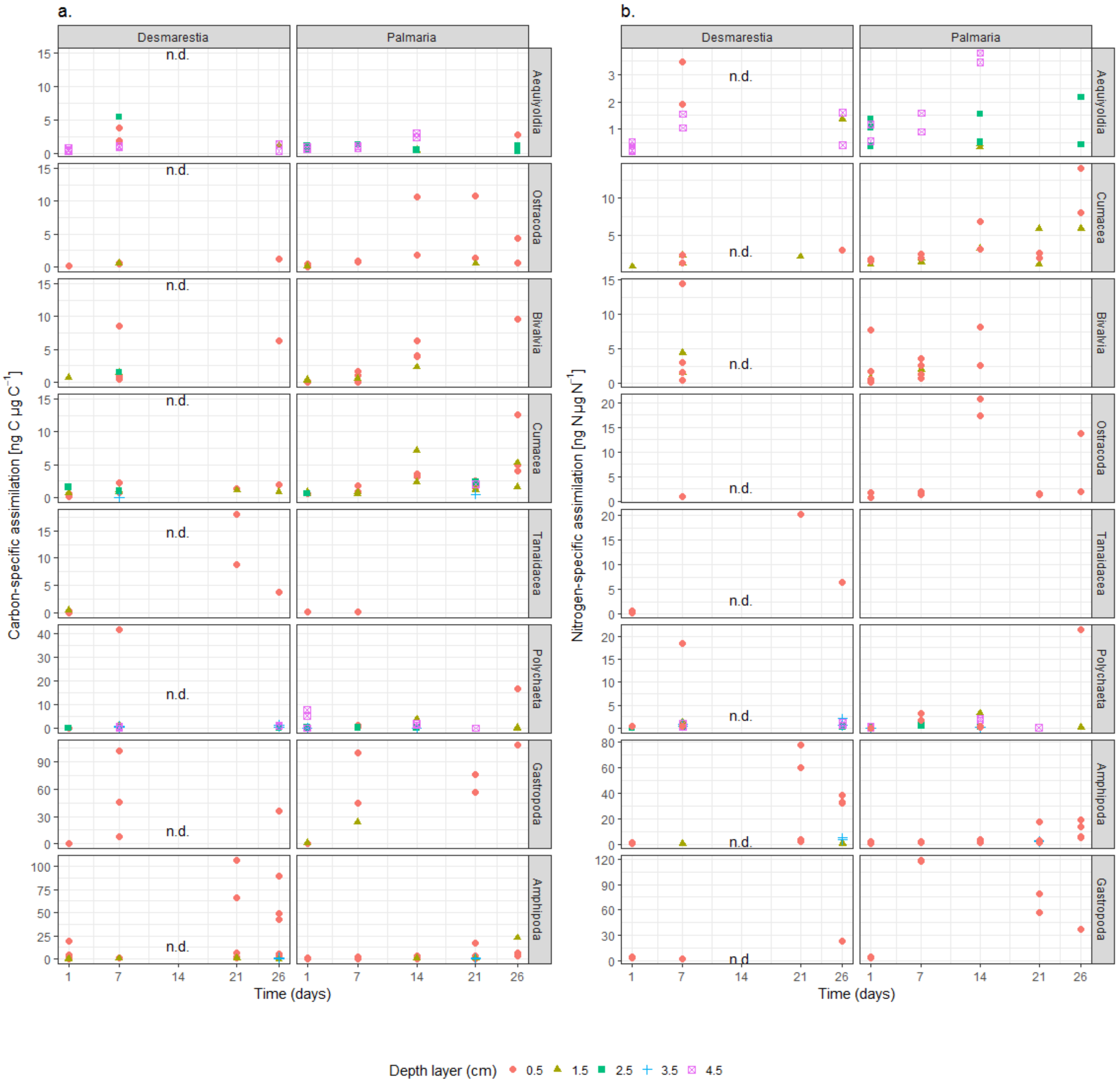

Figure S8: Specific assimilation of (a) carbon and (b) nitrogen by macrofauna in the macroalgae treatments over time. Plots are ordered according to increasing maximum assimilation.
